# Intermittent ozone inhalation during house dust mite-induced sensitization primes for adverse asthma phenotype

**DOI:** 10.1016/j.redox.2024.103330

**Published:** 2024-08-28

**Authors:** Salik Hussain, Nairrita Majumder, Md Habibul Hasan Mazumder, Sara E. Lewis, Olanrewaju Olapeju, Murugesan Velayutham, Md Shahrier Amin, Kathleen Brundage, Eric E. Kelley, Jeroen Vanoirbeek

**Affiliations:** a Department of Physiology, Pharmacology and Toxicology, School of Medicine, West Virginia University, Morgantown, WV, USA; b Center for Inhalation Toxicology (*iTOX*), School of Medicine, West Virginia University, Morgantown, WV, USA; c Microbiology, Immunology and Cell Biology, School of Medicine, West Virginia University, Morgantown, WV, USA; d Department of Biochemistry and Molecular Medicine, School of Medicine, West Virginia University, Morgantown, WV, USA; e Pathology, Anatomy and Laboratory Medicine, School of Medicine, West Virginia University, Morgantown, WV, USA; f KU Leuven, Department of Public Health and Primary Care, Centre for Environment and Health, Leuven, Belgium

**Keywords:** Lung, Inflammation, Ozone, T_H_17, Airway hyperresponsiveness, Lung function

## Abstract

The ability of air pollution to induce acute exacerbation of asthma is well documented. However, the ability of ozone (O_3_), the most reactive gaseous component of air pollution, to function as a modulator during sensitization is not well established. C57BL/6 J male mice were intranasally sensitized to house dust mite (HDM) (40 μg/kg) for 3 weeks on alternate days in parallel with once-a-week O_3_ exposure (1 ppm). Mice were euthanized 24 h following the last HDM challenge. Lung lavage, histology, lung function (both forced oscillation and forced expiration-based), immune cell profiling, inflammation (pulmonary and systemic), and immunoglobulin production were assessed. Compared to HDM alone, HDM + O_3_ leads to a significant increase in peribronchial inflammation (p < 0.01), perivascular inflammation (p < 0.001) and methacholine-provoked large airway hyperreactivity (p < 0.05). Serum total IgG and IgE and HDM-specific IgG1 were 3–5 times greater in HDM + O_3_ co-exposure compared to PBS and O_3_-exposed groups. An increase in activated/mature lung total and monocyte-derived dendritic cells (p < 0.05) as well as T-activated, and T memory lymphocyte subset numbers (p < 0.05) were noted in the HDM + O_3_ group compared to HDM alone group. Concurrent O_3_ inhalation and HDM sensitization also caused significantly greater (p < 0.05) lung tissue interleukin-17 pathway gene expression and mediator levels in the serum. Redox imbalance was manifested by impaired lung antioxidant defense and increased oxidants. O_3_ inhalation during allergic sensitization coalesces in generating a significantly worse T_H_17 asthmatic phenotype.

## Highlights

• Intermittent O_3_ inhalation primes for aggravated asthmatic responses.
• O_3_ aggravates HDM-induced lung inflammation and mucin expression.
• O_3_ co-exposure aggravates HDM-induced mixed Th2/Th17 phenotype.
• O_3_ and HDM co-exposure increases the expression of DC maturation (CD80, Dectin1) and migration (CCR7) markers.

## Graphical abstract

**Figure:**
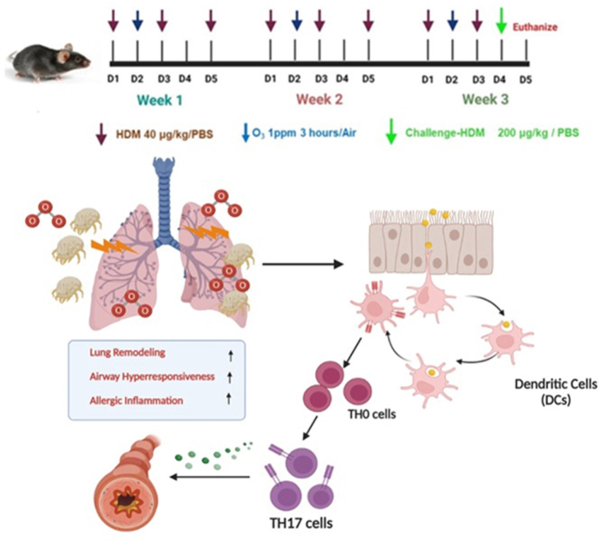

## Abbreviations

AHR =: Airway Hyperresponsiveness
Arg-1 =: Arginase 1
BALF =: Brochoalveolar Lavage Fluid
CCL2 =: C–C Motif Chemokine Ligand 2
CCR7 =: Chemokine Receptor 7
cDC =: Conventional Dendritic Cells
Col3a1 =: Collagenase 3a1
DC =: Dendritic Cell
DEP =: Diesel Exhaust Particles
ELISA =: Enzyme Linked Immunosorbent Assay
FEV_0.1=_: Forced Expiratory Volume
FOT =: Forced Oscillation Technique
FV =: Flow-Volume
GPX-4 =: Glutathione Peroxidase 4
H&E =: Hematoxylin and Eosin
HDM =: House Dust Mite
IFN-γ =: Interferon gamma
IL-10 =: Interleukin 10
IL-13 =: Interleukin-13
IL-17 =: Interleukin 17
IL-1β =: Interleukin-1ꞵ
IL-21 =: Interleukin 21
IL-22 =: Interleukin 22
IL-23 =: Interleukin 23
IL33 =: Interleukin 33
IL-4 =: Interleukin-4
IL-6 =: Interleukin-6
KC =: Keratinocyte chemoattractant
LPS =: Lipopolysaccharide
moDC =: Monocyte Derived Dendritic Cells
Muc5ac =: Mucin 5ac
Muc5b =: Mucin 5 b
NPFEE =: Negative Pressure Forced Expiration Extension
PC =: Provocative Dose
PCR =: Polymerase Chain Reaction
PM_2.5_ =: Particulate Matter_2.5_
PV =: Pressure-volume
QP3 =: Quick Prime 3
SD =: Standard Deviation
SEM =: Standard Error of Mean
TGF-ꞵ =: Transforming growth factor beta
T_H_17 =: T helper 17
T_H_2 =: T helper 2
TLR4 =: Toll Liker Receptor 4
TNF-α =: Tumor Necrosis Factor Alpha
TSLP =: Thymic Stromal Lymphopoietin
XOR =: Xanthine Oxidoreductase
α-SMA =: Alpha Smooth Muscle Actin

## 1 Introduction

Exposure to air pollution from various sources is associated with asthma development [1,2]. Living near major highways is associated with a greater risk of asthma in children with no family history of atopy [3]. Short-term exposure to air pollutants such as PM_2.5_, NO_2_ and O_3_ is positively associated with asthma mortality [4]. Moreover, asthmatics with allergic comorbidity are more susceptible to the ambient PM_2.5_ and O_3_ [5], yet experimental evidence of O_3_ as an adjuvant for asthma development is just beginning to emerge [[6], [7], [8], [9]]. Some of the most notable findings about allergic response development to HDM and O_3_ co-exposure come from studies on non-human primates (infant monkeys) as a model of childhood asthma and indicate the potential of co-exposure to alter lung development, increased airway responsiveness (on tissue slices), the roles of structural and functional localization of airways, epithelial innervation, and neuroendocrine cells [[10], [11], [12], [13], [14], [15]]. Air pollution particulates such as diesel exhaust particles (DEP) and PM_2.5_ are known to mediate allergy adjuvant responses [[16], [17], [18], [19]]. These particulate exposures are complex mixtures of elemental and organic carbon, endotoxin, metals, and aromatic hydrocarbons, which can have a significant impact of their own in shaping allergic responses. Nitrogen dioxide (5–70 ppm) and O_3_ (5–10 ppm) promote ovalbumin allergy at relatively elevated concentrations [18,20]. In Rhesus monkeys, repeated episodes of O_3_ inhalation result in greater allergic sensitization and airway immune and structural remodeling which causes airway hyperresponsiveness (AHR) by a serotonin-mediated pathway [15,21]. O_3_ exposure in an established asthma model decreased the levels of percutaneous oxygen saturation and increased inflammatory responses [22]. In humans, long-term O_3_ exposure is associated with adult-onset asthma but not with childhood asthma (reviewed in Ref. [23]). Single inhalation co-exposure to house dust and O_3_ induce a decrease in circulating endothelial progenitor cells (a risk factor for cardiovascular disease) and enhanced oxidative stress and certain inflammatory markers [24]. The impact of concurrent O_3_ exposure on asthma development during HDM sensitization is not thoroughly understood. The need to evaluate the potential role of O_3_ in asthma pathogenesis is further evident from the projections of increases in global O_3_ levels over the next decade.

Asthma is a heterogeneous chronic inflammatory disorder of the airways. It is defined by a history of respiratory symptoms (wheezing, cough, shortness of breath and chest tightness) with variable airflow obstruction [25]. Significant morbidity (5.8–8.4 %, ∼25 million individuals) and economic impacts (more than 5.8 million office visits) are reported for asthma in the United States [26]. Allergic sensitization to aeroallergens such as house dust mite (HDM) represents one of the most common etiologies for allergic asthma in the US [27]. *Dermatophagoides pteronyssinus* is the most common species of HDM associated with human allergy development. HDM-induced immune responses were initially described as T helper 2 (T_H_2) cells and B lymphocyte-mediated, but later contributions from the bronchial epithelium and innate immune mechanisms were recognized [28]. It is estimated that 40–70 % of asthma-associated hospital costs are related to the management of severe corticosteroid-insensitive asthmatic subjects [29]. Severe asthma is characterized by a mixed T_H_2/T_H_17 phenotype comprising eosinophils and neutrophils aggravated by environmental exposures [19]. It has already been proposed that different endotypes of asthma potentially develop due to the perturbation of distinct immunological pathways during sensitization [30].

The study herein is unique in the aspect that a low and environmentally relevant allergen exposure coupled with a single weekly O_3_ exposure was employed to evaluate the ability of the concurrent administration on the development of pulmonary inflammation, asthma phenotype, and modulation of immune response (T_H_17) locally as well as systemically. Further, this study provides detailed lung function assessments in intact animals that include translation-relevant forced expiratory measurements and calculation of methacholine provocative concentrations similar to those performed in clinics.

## 2 Experimental

### 2.1 Murine model

C57BL/6 J male mice (8 weeks old) were purchased from The Jackson Laboratory (Bar Harbor, ME) and acclimated at an AAALAC-accredited animal care facility at West Virginia University for seven days before exposure. All animal procedures were approved by the Institutional Animal Care and Use Committee (IACUC). The animals were monitored for weight change during the entire study.

### 2.2 Experimental design

**Fig. 1:**
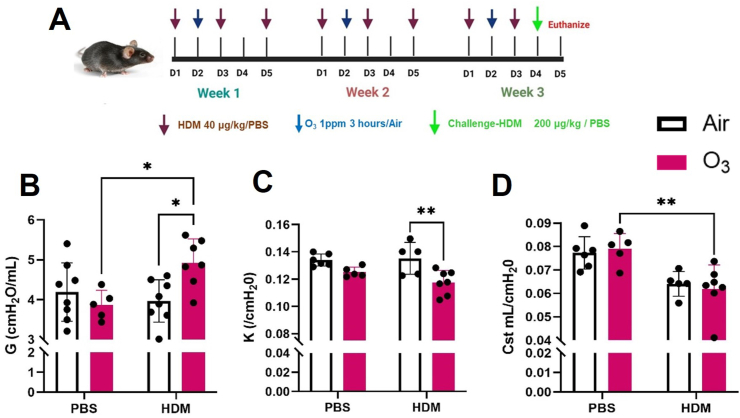
**House dust mite and ozone co-exposure induce lung function changes at baseline.** A) Experimental design. Baseline measurements of B) tissue damping (G), C) form of the deflating loop of the PV-Loop (K), and D) static compliance (Cst). Data are presented as mean ± SD of n = 5–8 mice per group and analyzed by Kruskal–Wallis test followed by Dunn's multiple comparison post hoc test. *P ≤ 0.05, **P ≤ 0.01.

The animals were randomly divided into four groups 1) PBS + Air 2) PBS + Ozone 3) HDM + Air 4) HDM + Ozone. For brevity these are mentioned as 1) Air, 2) Ozone, 3) HDM, 4) HDM + Ozone in the text and figures. Intranasal exposure to purified Dermatophagoides pateronyssinus extract from crushed whole bodies (XPB70D3A25, Greer Laboratories, Lenoir, NC) was performed. Exposure was standardized to the Der p1 (allergen) dosage for these studies and animals were exposed at a dose of 40 μg/kg for sensitization and 200 μg/kg for challenge (Fig. 1A). Exposures were performed during the work week (Monday-Friday). Animals were exposed to filtered air or ozone for 3 h at 1 ppm once weekly for three weeks. The mice were challenged with an intra-tracheal dose of house dust mite (200 μg/kg) 24 h before the euthanasia. The O_3_ was generated by passing pure oxygen through an ozone generator (HTU500AC, Ozone Solutions, Hull, IA) as described by us previously [31]. The animals were euthanized using intraperitoneal injection of Fatal Plus® at a dose of 250 mg/kg.

### 2.3 Bronchoalveolar lavage (BALF)

BALF was collected, and total and differential cell counts were performed as described previously [31]. The lavage supernatant was stored at −80 °C for further investigation. Lavage protein content was quantified using Pierce BCA Assay Kit (Thermofisher Scientific) as per the manufacturer's instructions.

### 2.4 Real-time PCR gene expression

Real-time PCR mRNA expression analyses were performed as described previously [31]. A list of Primers used for these assays can be found in Supplementary Table 1. Total RNA was isolated from unlavaged flash-frozen lung tissue using RNeasy Mini Kit (Qiagen, Germany) and reverse transcription was performed using High-Capacity cDNA Reverse Transcription Kit (Thermofisher Scientific). The Real-time PCR was performed as described previously [32] using AriaMx Real-time PCR System.

### 2.5 Lung histology

The non-lavaged lungs were fixed by intra-tracheal instillation of 10 % neutered buffered formalin and stained with hematoxylin and eosin (H&E) and Alcian Blue/PAS for routine light microscopic evaluation by a clinical pathologist in a blinded manner.

### 2.6 Lung function analysis

The lung function measurements were performed using Scireq Flexivent mechanical ventilator system (SCIREQ, Inc., Montreal, Canada) equipped with FX2 module and a negative pressure forced expiration (NPFEE) extension. Animals were anesthetized with urethane (2 mg/kg) and tracheotomized using a metal tracheal cannula (18 gauge, 03 cmH_2_O.s/mL resistance) and mechanically ventilated at 150 breaths/min, tidal volume of 10 mL/kg and positive end-expiratory pressure (PEEP) of 3 cmH_2_O with a computer-controlled ventilator system. The baseline lung function was measured after two deep inflations (30 cmH_2_0 pressure), by applying a broadband forced oscillation waveform (matched to the animal breathing frequency). The lung hyperresponsiveness was measured by exposing each mouse for 2 s to a solution of methacholine in increasing concentrations (0, 3.1, 6.25, 12.5, 25, 50 and 100 mg/mL) using the small particle size nebulizer (Aeroneb Lab nebulizer, 2.5–4 μm; Aerogen, Galway, Ireland). The nebulizer activation was synchronized with inspiration and set to a 50 % duty cycle for 5 s. The overall resistance and elastance were measured using the Snapshot 150 perturbation and fitted to a single-compartment model for quantitating the Respiratory system resistance (Rrs) and compliance (Crs). The Quick-prime 3 perturbations were run at 15 s apart five times for each dose of methacholine. The dose-response curve to methacholine was constructed in a cumulative manner. Each of the sequences was followed by a NPFEE measurement by inflating the lungs to 30 cmH_2_O over 1.2 s followed by immediate exposure of the animal's lungs to a negative pressure of −55 cmH_2_O to generate a negative expiratory pressure gradient. The NPFEE measurements were used to quantify the Forced Expiratory Volume at 0.1s (FEV_0.1_) for each of the methacholine doses. The provocative dose 30 (PC_30_) for FEV 0.1 was calculated from the slope of the dose-response curve of individual subjects. The Rn_100_ and Rrs_100_ were calculated for the provocative dose of the methacholine that would double the baseline responses in HDM + Air and HDM + O_3._ Small airway resistance and reactance were calculated as the difference in resistance between the lowest and highest frequencies tested in the prime wave-8 broadband forced oscillation maneuver, as described previously [33].

### 2.7 Cytokine/chemokine assays

Enzyme-linked immunosorbent Assay (ELISA) assays were performed on BALF and lung homogenate using Duoset sandwich ELISA assay kits (R&D Systems, MN) according to the manufacturer's recommendations. This assay was used to detect IL-33, TSLP, IL-4, INF-γ, TSLP, TNF-α, KC, IL-13, IL-6, TGF-ꞵ, IL-17 and IL-10. The concentration of different cytokines in serum were analyzed using mouse U-Plex Th-17 plex (combo 1) (K15077K; Meso Scale Diagnostics, Rockville, Maryland, United States) as per manufacturer's protocol. This kit included IL-17 A, IL-17C, IL-17 E/IL-25, IL-17F, IL-21, IL-22, IL-23, IL-31, and IL-33. The concentrations were generated as “calculated concentration means” on the MSD Discovery Workbench 4.0 software. All standards were run in duplicates.

### 2.8 Immunoglobulin assays

Total serum IgG level was measured using the IgG Mouse ELISA Kit (Invitrogen), as per the manufacturer's instruction. HDM-specific IgG1 levels were quantified by coating the plates overnight with house dust mite at a concentration of 2 μg/mL at 4 °C. The plates were washed with wash buffer and blocked with blocking buffer for 2 h at room temperature with constant shaking. This was followed by washing and the addition of samples (diluted to 1:2000 using assay buffer) followed by incubation at RT for 2 h. The plates were washed and IgG1 specific antibody (The Jackson Immuno Research, Catalog# 115-065-205) was added at a concentration of 1:40,000 with incubation for 2 h with constant shaking. The plates were washed and incubated with HRP for 30 min followed by the addition of substrate reagent and reading at 450 nm wavelength in a microplate reader. Serum total IgE was measured using the Total IgE ELISA Max Kit (BioLegend) per the manufacturer's instruction. HDM-specific IgE level was quantified by coating the plate overnight with 6 μg/mL house dust mite at 4 °C. This was followed by washing the plate with wash buffer and blocking for 1 h at RT. The plates were washed and diluted samples (1:2000) were added and incubated for 1.5 h at RT followed by wash. The detection antibody (Southern Biotech, Catalog #1110–08) was added at a dilution of 1:2000 and plates were incubated for 1.5 h at RT. The plates were washed, HRP treated and read with substrate reagent at 450 nm wavelength using a microplate reader.

### 2.9 Immune cell phenotyping

Lung lymphocytes and dendritic cells were identified using flow cytometry. Lung tissue was dissociated in an enzymatic digestion buffer [34]. Lung tissues were minced and incubated at 37 °C to activate enzymatic reaction. Homogenized lung tissues were neutralized by adding DMEM (Dulbecco's Modified Eagle Medium) containing 10 % FBS (fetal bovine serum) and passed through 100 μM nylon filters for single-cell suspension. Red blood cells were lysed using BD Pharm Lyse™ (BD Biosciences, San Jose, CA). Cells were counted using a countess cell counter (Invitrogen, Carlsbad, CA). Cells were stained with viability dye, preincubated with FcRBlock (Miltenyl Biotec, Germany), and labeled with combinations of fluorochrome-conjugated monoclonal antibodies. Cytofix/Cytoperm fixation/permeabilization kit (BD Biosciences, San Jose, CA) was used before labeling with FoxP3. The gating strategy for lymphocytes is shown in Supplementary Fig. 7A. Cells were gated into B cells, T cells, memory cells, T helper cells, cytotoxic T cells, activated T cells and regulatory T cells. The cell surface and intracellular markers used to identify lymphocytes are described in Supplementary Table 2. The gating strategy for identifying dendritic cells is shown in Supplementary Fig. 7B. DCs were gated into plasmacytoid DC (pDC), type 1 conventional DC (cDC1), type 2 conventional DC (cDC2), and monocyte-derived DC (moDC). Activation status and maturation of the dendritic cell population were identified using CD80, Dectin1 and CCR7. Markers for DC phenotyping are described in Supplementary Table 3. The gating strategy for these markers is shown in Supplementary Fig. 7C. Data were acquired on the BD LSR Fortessa II using BD FACSDiva software. Analysis was performed using FCS Express 7 (De Novo Software, Glendale, CA).

### 2.10 Western blot protein expression

Flash-frozen lung tissue was pulverized at −80 °C and lysed using RIPA buffer (Thermo Fisher Scientific) supplemented with protease and phosphatase inhibitor cocktail (Thermo Fisher Scientific). Total protein content was analyzed using the BCA (bicinchoninic acid) assay (Thermo Fisher Scientific). The proteins were separated using 4–12 % bis-tris polyacrylamide gel transferred to PVDF membrane. The membranes were blocked using 5 % milk and incubated overnight at 4 °C with 1:500 dilution of rabbit polyclonal antibody (cat#10005258 GPX4 Polyclonal Antibody, Cayman Chemical). Membranes were washed with tris buffered saline tween solution and conjugated with anti-rabbit HRP conjugated secondary antibody for 1 h (1:10,000) (Cell Signaling Technologies). The membranes were developed with ECL (ThermoFisher Scientific) and detected using the Amersham Imager 600 (Cytiva, Life Sciences, Marlborough, MA) imaging system. β-actin was used as a housekeeping control at a dilution of 1:1000 (catalog # sc-47778, SantaCruz).

### 2.11 Redox measurements

#### 2.11.1 Hydrogen peroxide level detection (amplex red assay)

Hydrogen peroxide H_2_O_2_ levels in the whole lung tissue and BALF were quantified using Amplex™ Red Hydrogen Peroxide/Peroxidase Assay Kit (Thermo Fisher Scientific, Waltham, MA), following manufacturer's instructions.

#### 2.11.2 Total glutathione assay

Flash-frozen lung tissues were pulverized at −80 °C and glutathione content in lung homogenate was quantified using the OxiSelect™ Total Glutathione (GSSG/GSH) Assay Kit, following the manufacturer's instruction.

#### 2.11.3 XOR activity assay

Lung homogenates were assessed for XOR activity as previously described using electrochemical detection (ESA Coul-Array System) of UA generation using reverse-phase high-performance liquid chromatography [32].

### 2.12 Statistical analyses

Data are presented as means ± standard deviation (SD) from at least two repeats. Animal numbers and raw data values used to generate the heatmap are presented in *Supplementary Excel File*. D'Agostino-Pearson or Shapiro-Wilk test was used to check normality (depending on group size). One-way or two-way analysis of variance (ANOVA) followed by Tukey's post hoc test was used for normal distributed data sets. In case of a failed normality test, a non-parametric Kruskal Wallis test followed by Dunns post hoc test for multiple comparisons was performed. Individual comparisons between groups were performed by the Student-t test or Mann-Whitney *U* test as appropriate. A two-tailed p-value of less than 0.05 (95 % confidence level) was considered statistically significant. Statistics were performed using GraphPad Prism v7.

## 3 Results

### 3.1 Adverse asthma phenotype-induction by O_3_ and HDM co-exposure

At baseline, a significant increase in tissue dampening (G) was observed in the HDM + O_3_ exposure group compared to HDM alone and O_3_ alone groups (Fig. 1B). Lung pressure-volume loops (PV- loop) derived deflating loop curvature (K) demonstrated a significant reduction in HDM + O_3_ compared to the HDM alone group (Fig. 1C) while static compliance (Cst) demonstrated a significant reduction in HDM + O_3_ compared to O_3_ alone groups (Fig. 1D). PV-loops are presented in Supplementary Fig. S1A. Furthermore, lung Impedance (Z) showed no differences between any groups at baseline (Supplementary Fig. S1B).

**Fig. 2:**
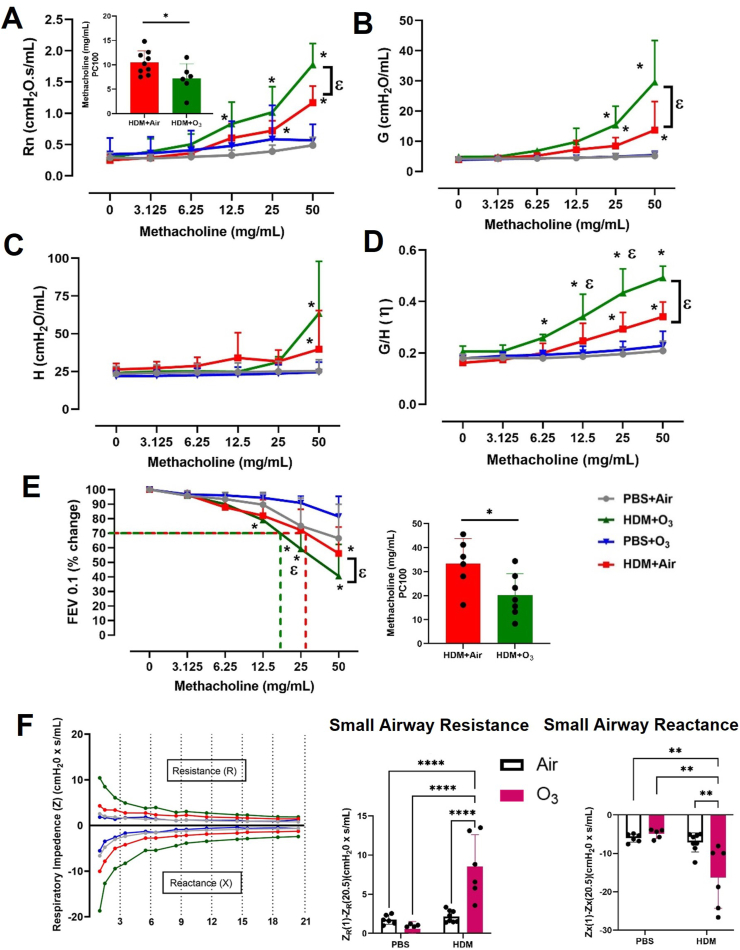
**House dust mite and ozone co-exposure induce airway hyperresponsiveness.** A) Methacholine dose response of Newtonian resistance (Rn) and provocative concentration 100 (PC_100_) calculation for HDM and HDM + O_3_ groups (inset) B) Methacholine dose response of tissue damping (G) C) Methacholine dose response of tissue elastance (E). D). tissue Hysteresivity (η) calculated as G/H E) % change in Forced Expiratory Flow at 0.1s (FEV_0.1_) after exposure to increasing methacholine doses and inset is provocative concentration 30 (PC_30_) calculation for FEV0.1. F) Impedance plots at 50 μg/mL methacholine with specific small airway resistance and reactance plotted as bar graphs. Respiratory impedance was measured using the forced oscillation technique with pseudo-random oscillations over a range of 1–20.5 Hz depicting the real (i.e., resistance) and imaginary (i.e., reactance) part of the impedance. Mice were exposed to PBS + Air, PBS + ozone (1 ppm; 3 h), HDM + air, HDM + ozone (1 ppm; 3 h). Data are presented as mean ± SD of n = 5–9 mice per group and analyzed by two-way analysis of variance (ANOVA) followed by Tukey's post hoc test or students t-test. *P ≤ 0.05 vs PBS + Air, ε P ≤ 0.05 between HDM + Air and HDM + O_3_ that methacholine concentration. For Impedance measurements *P ≤ 0.05, **P ≤ 0.01, ***P ≤ 0.001, ****P ≤ 0.0001.

After the baseline measurements, we performed a methacholine provocation/challenge protocol and observed a significantly greater large airway hyperreactivity (Rn) in the case of O_3_ and HDM co-exposure (Fig. 2A). This was further confirmed by the significantly lower methacholine concentration required to double the Rn (PC_100_ values) (inset Fig. 2A). Tissue Damping (G) and tissue Elastance (H) demonstrated similar hyperresponsive patterns (Fig. 2B and C). Lung tissue hysteresivity (G/H, η) further demonstrated significant increases in HDM + O_3_ group starting at methacholine doses as low as 6.25 mg/mL (Fig. 2D). A significant decrease in forced expiratory flow in 0.1 s (FEV_0.1_) and provocative concentration of methacholine to induce a 30 % decrease (PC_30_) further confirmed worse obstructive phenotype in HDM + O_3_ group compared to the HDM alone group (Fig. 2E). Lung tissue impedance (Z) analyses also demonstrated significant hyperresponsiveness in the small airways after methacholine provocation as indicated by an increase in small airway resistance and reactance (Fig. 2F). A significant increase in total respiratory resistance (Rrs) and a significantly lower PC_100_ value further confirmed respiratory hyperresponsiveness (Supplementary Figure 2). The airway obstructive pattern was further confirmed by the aggravated obstructive patterns seen on Flow-volume (FV) loops and time-volume loops (Supplementary Figure 3).

### 3.2 Aggravation of pulmonary inflammation and remodeling by O_3_ and HDM co-exposure

**Fig. 3:**
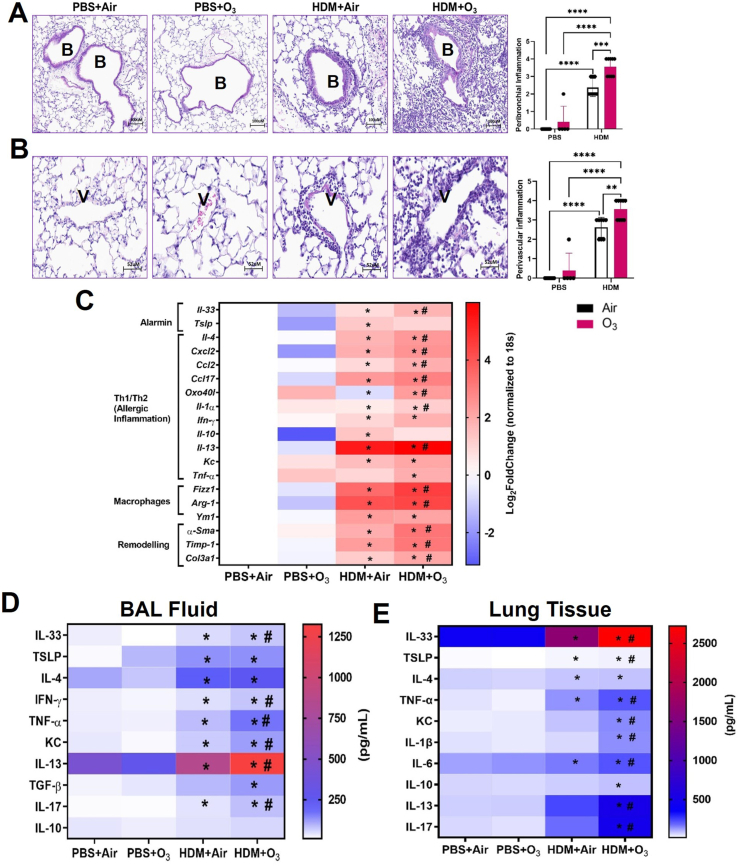
**House dust mite and ozone co-exposure induce lung inflammation** A) Peribronchial Inflammation B) Perivascular Inflammation. n = 5–9 mice per group and analyzed by Kruskal–Wallis test followed by Dunn's multiple comparison post hoc test. *P ≤ 0.05, **P ≤ 0.01, ***P ≤ 0.001, ****P ≤ 0.0001C). Lung tissue real-time PCR analyses for mRNA expression D) BAL fluid ELISA E) Lung homogenate ELISA. n = 4–7 mice per group and analyzed by two-way analysis of variance (ANOVA) followed by Tukey's post hoc test. PCR values are presented as Log 2-fold change values. * Represents significantly different from PBS + Air while # represents significantly different between HDM + Air and HDM + O_3._

Exposure to HDM + O_3_ induced a significantly greater inflammatory cell influx around the airways and blood vessels in the lungs compared to HDM extract alone (Fig. 3A and B). The infiltrating cell population is comprised of a mixture of macrophages, lymphocytes, neutrophils, and eosinophils. These findings were validated by histopathological scoring performed by a pathologist in a blinded manner. BALF total and differential cell counts confirmed a significant increase in total cells, lymphocytes, eosinophils, and neutrophils by HDM and HDM + O_3_ (Supplementary Fig. 4A). However, there was no difference between the HDM alone or HDM + O_3_ groups. A comparable increase in BALF total proteins indicated a similar change in lung permeability by the HDM alone or HDM + O_3_ groups (Supplementary Fig. 4B).

Co-exposure to HDM and O_3_ induced a significantly greater gene expression of alarmin (*Il-33*), Th2 cytokines (*Il-4, Il-13*), M_2_ macrophage markers (*Fizz 1, Arg-1*) and remodeling genes (*Timp-1, Col3a1, α-Sma*) in the lung tissue (Fig. 3C). Allergic inflammation-related cytokines (*Ccl2, Ccl17*) mRNA expression was significantly greater after HDM + O_3_ than the HDM alone. Apart from *Tnf-α* and *Cxcl2* which were significantly induced by HDM + O_3_, Th1 cytokines (*Ifn-γ*, *Kc*) and M2 macrophage marker *Ym-1* were induced similarly between HDM alone and HDM + O_3_. In addition, *Il-5, Gm-csf, Ox40, Il-31r and Stat-1* were induced similarly between HDM alone and HDM + O_3_ (Supplementary Fig. 4C). *Tslp* and *IL-10* were only induced in the HDM alone group. BALF ELISA further demonstrated significantly greater protein levels for IL-33, IFN-γ, TNF-α, KC, IL-13 and IL-17 in the HDM + O_3_ exposed mice, while TSLP and IL-4 levels were elevated to a similar extent between HDM alone and HDM + O_3_ groups. Lung tissue homogenate ELISA also confirmed the modulatory abilities of O_3_ in terms of multiple cytokines (TSLP, IL-33, IL-17, IL-13, IL-10, IL-6, KC, IL-1ꞵ and TNF-α), while IL-4 increase was similar between HDM and HDM + O_3_ groups and IL-10 was only induced in HDM + O_3_ group (Fig. 3E).

**Fig. 4:**
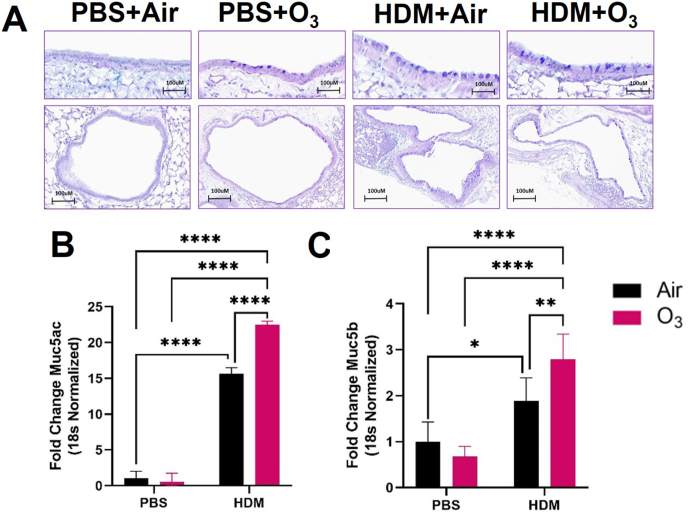
**House dust mite and ozone co-exposure induce mucous cell metaplasia and mucin gene expression.** A) histopathological evaluation of mucous cell metaplasia by AB/PAS staining B) Real-time PCR analyses for Muc5ac and C) Muc5b gene expression in lung tissue. Data are presented as the mean ± deviation (SD) and analyzed by two-way analysis of variance (ANOVA) followed by Turkey's post hoc test. n = 4–6 animals per group. *P ≤ 0.05, **P ≤ 0.01, ****P ≤ 0.0001.

Lung tissue remodeling and increased mucous production are common features of obstructive pulmonary disorders. We further observed significant mucous cell metaplasia (MCM) on histological sections from HDM and HDM + O_3_ co-exposure groups (Fig. 4A). Real-time PCR-based mRNA expression analyses confirmed significantly greater mucin gene expression (*Muc5ac* and *Muc5b*) in the HDM + O_3_ group compared to HDM alone (Fig. 4B and C).

### 3.3 Altered immune phenotype after O_3_ and HDM co-exposure

**Fig. 5:**
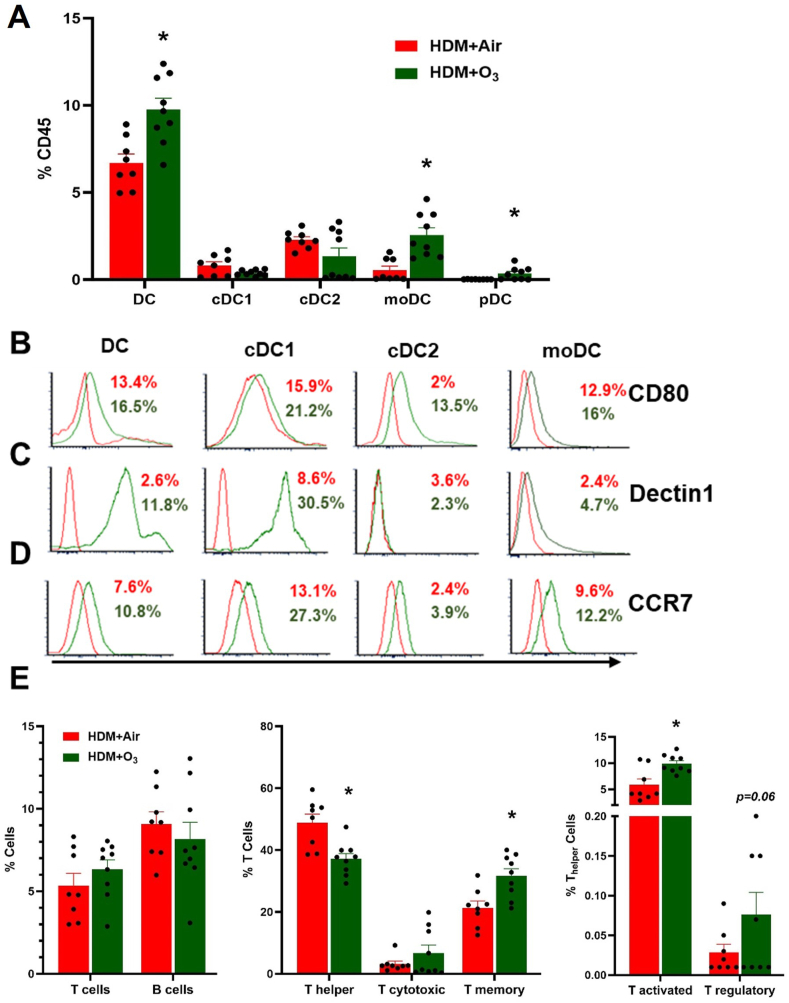
**Flow cytometry analyses of lung immune cells.** A) Lung dendritic cells (total DC, moDC, cDC1, cDC2 and pDC) are presented as % of CD45 (+) cells in HDM and HDM + O_3_ groups. Activation of DC populations. B) CD80 C) Dectin 1 and D) CCR7 expression on lung dendritic cells. after HDM or HDM + O_3_ exposure. E) Alterations in lung lymphocyte subpopulations. n = 8–9 mice per group. Data are presented as mean ± SEM, Mann-Whitney test was performed. * denotes the significance between HDM and HDM + O_3_ at P ≤ 0.05.

HDM + O_3_ exposure dysregulated immune cell profile to a significantly greater extent compared to HDM or O_3_ alone. A significantly greater increase in total lung DCs, moDCs, and pDCs was noted in HDM + O_3_ compared to HDM alone group (Fig. 5A). O_3_ exposure did not further increase the numbers of cDC1 and cDC2 subsets of conventional DCs. In general, more DCs (all subsets) demonstrated maturation marker CD80 in the case of O_3_ and HDM co-exposure compared with HDM alone (Fig. 5B). Co-exposure increased Dectin-1 positive total DC and cDC1 and moDCs (Fig. 5C). More CCR7-positive cells were noted in all DC populations indicating an increased proportion of migratory cells (Fig. 5D). Lung T and B lymphocyte populations were similarly elevated in HDM and HDM + O_3_ groups while intriguingly, Th cells showed a decrease, and T memory cells showed an increased percentage in the HDM + O_3_ group compared to the HDM alone group (Fig. 5E). While the percentage of Th cells demonstrated a decrease the absolute numbers were not altered (Supplementary Fig. 5). Among T cell subsets, there was an increase in activated T cells with a trend toward an increase in T reg cells (p = 0.06) (Fig. 5E).

### 3.4 O_3_ and HDM co-exposure induces greater immunoglobulin production

**Fig. 6:**
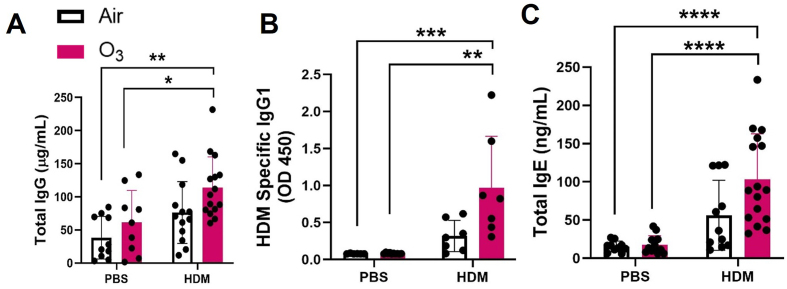
**HDM and O**_**3**_**co-exposure induces the production of serum IgG and IgE.** A) Total IgG in mouse serum (1:50,000 dilution) B) HDM specific serum IgG1 (1:2000) C) Total IgE in mouse serum (1:50). Data are presented as mean ± SEM. n = 9–10 mice per group. Kruskal–Wallis test followed by Dunn's multiple comparison post hoc test. * represents significantly different (P ≤ 0.05) from PBS + Air, # presents significantly different (P ≤ 0.05) between HDM + Air and HDM + O_3_.

An increased immunoglobin production in terms of total *IgG* and HDM specific *IgG1* (Fig. 6A and B) and total *IgE* (Fig. 6 C) were detected in the serum from HDM + O_3_ exposed mice while no significant alteration in HDM specific *IgE* was noted (Supplementary Fig. 6).

### 3.5 Increased Th-17 pathway activation by HDM and O_3_ co-exposure

**Fig. 7:**
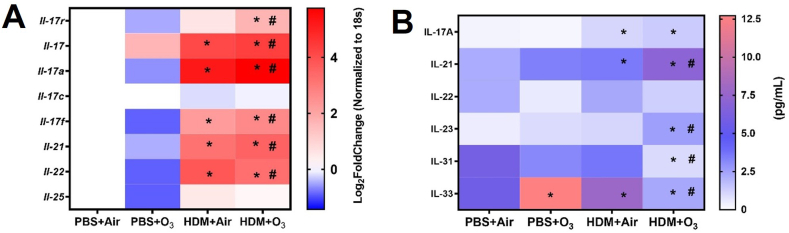
**T-helper 17 induced signaling pathway** A) Lung tissue real-time PCR mRNA expression. B) Serum ELISA for TH-17 pathway mediators. PCR values are presented as Log 2-fold change values. Data are presented as mean ± SD of n = 4–6 mice per group and analyzed by Two-way analysis of variance (ANOVA) followed by Tukey's post hoc test. * represents significantly different (P ≤ 0.05) from PBS + Air while # presents significantly (P ≤ 0.05) different between HDM + Air and HDM + O_3._

A significantly greater lung tissue gene expression of IL-17 pathway genes (*Il-17r, Il-17, Il-17a, IL-17c, Il-17f, Il-21 and, Il-22* was observed in the HDM + O_3_ exposure compared to HDM alone (Fig. 7A). Serum levels of IL-17 A, IL21, IL-23, IL-31 and IL-33 were also significantly elevated in the HDM + O_3_ exposure compared to HDM alone (Fig. 7B).

### 3.6 O_3_ and HDM co-exposure induces greater redox imbalance

**Fig. 8:**
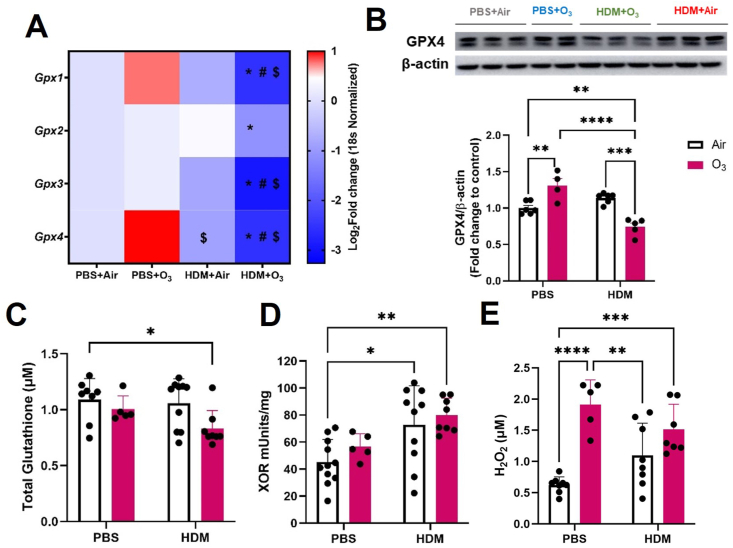
**Ozone and house dust mite-induced redox imbalance** A) Lung tissue real-time PCR analyses (mRNA expression), B) Western Blot analysis of GPX4 expression in lung homogenate. C) Glutathione concentration in lung tissue homogenate. D) Xanthine oxidoreductase activity in lung tissue homogenate. E) H_2_O_2_ levels in BAL fluid. PCR values are presented as Log 2-fold change values. Data are presented as mean ± SD n = 4–10 mice per group and analyzed by Two-way analysis of variance (ANOVA) followed by Tukey's post hoc test. *P ≤ 0.05, **P ≤ 0.01, P ≤ 0.001, ****P ≤ 0.0001.

Co-exposure to O_3_ and HDM induced a unique redox profile as indicated by gene expression downregulation of glutathione peroxidase (*Gpx 1-4*), reduction of GPX4 protein levels, and decrease in total glutathione (GSH) levels in the lungs (Fig. 8A–C). This was accompanied by an increase in xanthine oxidoreductase (XOR) activity in the lung tissue and a significant increase in H_2_O_2_ levels in the BALF (Fig. 8D–E).

## 4 Discussion

Herein we demonstrate the capacity of O_3_ to aggravate HDM allergen exposure generated mixed T_H_2/TH17-predominated severe asthma phenotype. This was evidenced by the increase in immunoglobulins (IgE, IgG1), airway hyperresponsiveness (Rn and FEV_0.1_), increase in peribronchial inflammatory cell accumulation, and increased gene and protein expression of different T_H_2/T_H_17 pathway members and mediators. This study mechanistically defines the asthmatic and inflammatory/remodeling parameters modulated by O_3_ and HDM allergen co-exposure. In this study, we have selected a recurrent/periodic O_3_ exposure that did not induce inflammation; however, when combined with exposure to HDM allergen it did potentiate the capacity of the allergen to induce asthmatic phenotype and skew the inflammation towards a T_H_17 predominant phenotype. Previously, it was demonstrated that while both O_3_ and HDM-mediated AHR require Natural killer cells only O_3_ exposure-induced AHR required NKT cells producing IL-17 [35].

Asthma involving a T_H_17 phenotype/neutrophilic asthma is often considered severe and in addition to AHR, it manifests as an increased IL-17 A production, neutrophilia, and resistance to corticosteroids [36,37]. Our results showing a significant upregulation of T_H_17 pathway related gene expression (*Il-17r, Il-17, Il-17a, Il-17f, Il-21, and Il-22*), protein expression in the lungs (IL33, IL-17), in the BALF (IL-17, IL-33) and in the serum (IL-17 A, IL-21, IL-23, IL-33) in the HDM + O_3_ exposed mice point towards skewing of immune response towards T_H_17 phenotype. IL-33 is known to mediate barrier disruption in the lungs after O_3_ exposure and lung barrier disruption is known to increase the allergen sensitization [38,39]. IL-33 orchestrates tissue remodeling and immune response in chronic airway inflammation through both T_H_2 (initiated by ILC2 matured by IL-33) and T_H_17 (via IL-1β and IL-6 derived from IL-33-matured DCs) immune responses [40]. IL-33 also plays a significant role in DEP-mediated aggravation of HDM-induced allergic inflammation [41]. Moreover, HDM + O_3_ exposure induced a significantly greater gene expression of Ccl17 that has pleiotropic roles in allergic inflammation/asthma in part through T_H_17 cell recruitment.

Intriguingly, we observed a decrease in the levels of IL-31 and IL-33 in the serum. Both IL-31 and IL-33 are typically considered inflammatory however, their context-dependent opposite impacts are also reported [[42], [43], [44]]. Functions of IL-33 are very well documented in the lungs during asthma [45,46] while systemic functions during allergic inflammation are not well described and are sometimes divergent [43,44]. IL-33 is usually secreted at the barrier tissue level to promote innate immune response. A significant increase in IL-33 and IL-13 expression levels in the lung tissue and lavage fluid in the HDM + O_3_ exposure group indicates a potential contribution to lung immune response potentially through multiple pathways including ILC2s and activation of various immune cells. Further, mechanistic studies are needed to establish the role of this pathway in O_3_-induced aggravation of HDM asthma.

DC express a variety of innate receptors that allow them to directly sense the immunologic stimuli and tailor the immune response [47]. Epithelium-derived factors (TSLP, IL-33, CCL2) are also known to induce DC migration and maturation, especially of moDCs in severe asthma [48]. We also observed a significant increase in the number of moDCs (CD45^+^, CD11c^+^, MHC2^+^, CD64^+^) in the lungs. Moreover, a significantly increased expression of CD80 indicated increased maturation/activation while CCR7 expression indicated an increased proportion of migratory cells as DC migration to the draining LNs is controlled by CCR7. Due to their proclivity of inflammatory cytokine production and antigen presentation to effector T cells, moDCs may differentially initiate or propagate T_H_2 responses to HDM [47]. Moreover, a significant increase in a C-type lectin receptor (Dectin-1) was observed in DC, cDC1 and moDC populations. Dectin-1 is expressed by multiple cell types in the lungs and was shown to be an integral DC subset for the development of allergic airway inflammation and T_H_17 cells after house dust mite exposure by recognizing ꞵ-glucans [49]. Dectin-1 also increases the activation and migration of CD11b^+^ DCs to lymph nodes by increasing the expression of various chemokines including CCR7 [49]. Interestingly, we observed Dectin-1 expression on a proportion of CD103^+^ DCs in addition to moDC's and their number further increased in the case of O_3_ co-exposure. In addition, Dectin-1 on DC subsets is also crucial for T-cell polarization and primes for cytotoxic T-cell responses [50].

Our lung function data provide significant insight into the phenotype of lung diseases in the case of HDM and O_3_ co-exposure. Increased AHR is a hallmark of asthma and a significant decrease in the provocative concentration of methacholine for large airway resistance (Rn), pulmonary resistance (Rrs) and airflow obstruction (FEV_0.1_) confirms aggravated asthmatic responses in O_3_ and HDM concurrent exposure. Increased baseline G, K and Cst as well as AHR in terms of Rn (PC_100_) and FEV_0.1_ (PC_30_) further confirm the aggravating impacts of O_3_ in pulmonary function decline. Moreover, our impedance (Z) results confirm a significantly greater small airway dysfunction (decreased small airway resistance and increased small airway reactance) after HDM + O_3_ exposure only in the case of methacholine provocation but not at the baseline. Moreover, FV-loop and flow-time curves further confirm the aggravated obstructive phenotype after methacholine provocation. Taken together, our data confirm both small and large airway-centric AHR and obstructive phenotype in the O_3_-mediated aggravation during HDM-induced lung disease.

Previous experimental studies on asthma adjuvant effects with gaseous components of air pollution were performed at high concentrations reaching unhealthy levels (5–70 ppm NO_2_, and 5–10 ppm O_3_) [18,20]. Resting rodents require 4–5 times the O_3_ dose to achieve similar deposition and reproduce biological outcomes as exercising humans in controlled exposure studies [51,52]. Based on this, our 1 ppm exposure in resting rodents translates to be less than O_3_ concentrations of 0.4 ppm that caused pulmonary neutrophilia in resting human adults receiving short-term exposures [51] or approximately comparable to O_3_ concentrations of 0.16–0.20 ppm that caused pulmonary function impairments in exercising adults receiving short-term exposures [53,54]. However, if considered without the above-mentioned factors, it is approximately 8- and 14-fold higher than the 1-h (0.12 ppm) and 8-h (0.070 ppm) national ambient air quality standard concentrations. HDM dose and composition are described as critical determinants for the type of allergic response [55,56]. We utilized repetitive exposure to a HDM (40 μg/kg sensitization and 200 μg/kg for challenge). As HDM contains other immunologically active ingredients such as LPS, proteases, chitins and ꞵ-glucans there is always the possibility of their involvement in the resulting immune and asthmatic phenotype development [55]. These also contribute to observed mixed eosinophil and neutrophil phenotype and AHR [55,57,58]. In addition, components of house dust can directly program lung conventional DC (and not moDC) to promote T_H_2 cytokine production independent of TLR4 signaling and epithelial-derived cytokines [47].

Redox imbalance has been widely reported in chronic respiratory disease pathogenesis by contributing to variety of hallmarks of these diseases such as mucus hypersecretion, airway hyperresponsiveness and remodeling. A decrease in peripheral blood glutathione peroxidase activity increased oxidant production, and an imbalance between oxidants and antioxidant levels has already been documented in asthmatic individuals [[59], [60], [61]]. These studies point towards the potential impairment of the antioxidant defenses in the respiratory tract during asthma. Acute glutathione depletion can augment airway hyperreactivity and inflammation in allergic mice [62]. In our animal model, we observed a significant downregulation of GSH/*Gpxs* and upregulation of oxidant production in the case of HDM + O_3_ co-exposure that further validated a modulatory impact of allergen and air pollution in a co-exposure model. The increased activity of xanthine oxidoreductase (XOR), a known source of oxidants, in the lungs further confirms the increased potency of HDM and O_3_ co-exposure to produce redox imbalance in the lungs compared to HDM or O_3_ alone exposures. XOR upregulation and involvement in HDM-induced asthma have been previously reported [63]. In this context, we also observed elevated H_2_O_2_ levels and diminished total glutathione levels in the same cohorts; however, our experiments were not designed to specifically assign this observation to XOR nor did they specify GSH vs. GSSH. We quantify a recognized source of peroxide to be elevated concomitant with increased rates of peroxide generation and diminution of total glutathione; the latter potentially being driven by generation of adducts to proteins that diminish the relative amounts of GSSG accessible to the assay. Given that redox imbalance is implicated in corticosteroid insensitivity in asthma and is known to mediate T_H_17 phenotypes, it is potentially possible that it has a role to play in the aggravating effects of O_3_ in HDM asthma [64,65].

The strengths of the present study include a model that more closely resembles human exposure by implementing a paradigm of low-dose and multiple HDM exposures with or without once-a-week O_3_ exposure. The detailed exploration of lung function indices and measurements of soluble factors as well as cellular outcomes provides additional strength. Limitations include the use of a single HDM lot and the lack of evaluation of the relative contribution of proteases or LPS in the assessed outcomes. The use of only male C57BL/6 J mice (a low T_H_2 responder strain) is also a limitation. However, as adult females develop more severe asthma, it is anticipated that the modulating impact of air pollution could be even more severe. In addition, another limitation of the current study is not identifying the source of IL-17 and the relative contribution of HDM components. Further in-depth evaluations would include the migration of DC's, Treg functionality, Th cells specificity for HDM, and cytokine expression in each subset to decipher the impact of O_3_ on each DC subset. For future studies, including ‘real-time’ lung function measurements just before, during, or directly after exposure to O_3_ and HDM extract, could identify early respiratory responses, possibly including direct obstructive asthmatic responses, which would add to the invasive measurements that we performed in this study. Furthermore, a more detailed exploration of the role of oxidants (sources and species) in this process is also required. For example, we presented herein only XOR activity, total glutathione content, and peroxide levels and did not pursue granularity regarding GSSG vs. GSH.

## 5 Conclusions

We provide evidence of severe asthmatic phenotype (airway hyperresponsiveness) coupled with increased lung inflammation/remodeling as well as greater T_H_2/T_H_17 milieu in an O_3_ and HDM co-exposure scenario. These studies further establish that periodic O_3_ exposure alone is not sufficient to develop allergic inflammation; yet, when combined with the allergen, it can potentiate the capacity of the allergen to induce an asthmatic phenotype and skew the immune response towards a T_H_17-predominant phenotype. Further studies with both sexes and potentially susceptible mouse strains (collaborative cross and allergy-prone) [66] would be needed to validate these findings and determine the exact working mechanism of the stimulatory O_3_ exposure. These results highlight the need to consider environmental exposures in addition to atopy as a contributing factor for human asthma development. The significance of these studies is further increased in the ongoing global climate crises (wildfires, increased heat events and raised humidity) contributing to potentially elevated levels of O_3_ in the environment. As such, this work further affirms the pressing need for global climate action to curb the levels of O_3_ and reinforces the need for more mechanistic insights to develop therapeutic interventions for environmental asthma.

## Funding statement

This study was supported by National Institute of Health funding R01 ES031253 (SH), ES031253-04S1 (SH) and R01 DK124510, R01 HL153532-01A1 (EEK). The WVU Flow Cytometry & Single Cell Core Facility (KB) is supported by NIH grants P20 GM121322, U54 GM104942, U54 GM103434, and S10OD016165. Funding sources had no role in any aspect of the study.

## Conflict of interest and financial disclosure

None.

## Statement of competing interests

The authors declare that they have no competing interests.

## CRediT authorship contribution statement

**Salik Hussain:** Writing – review & editing, Writing – original draft, Supervision, Resources, Project administration, Methodology, Funding acquisition, Conceptualization. **Nairrita Majumder:** Visualization, Methodology, Investigation, Formal analysis. **Md Habibul Hasan Mazumder:** Visualization, Software, Methodology, Investigation, Formal analysis, Data curation. **Sara E. Lewis:** Methodology, Investigation, Formal analysis. **Olanrewaju Olapeju:** Visualization, Methodology, Investigation, Formal analysis. **Murugesan Velayutham:** Visualization, Methodology, Investigation, Formal analysis. **Md Shahrier Amin:** Writing – review & editing, Methodology, Investigation, Formal analysis, Data curation. **Kathleen Brundage:** Methodology, Investigation, Formal analysis, Data curation. **Eric E. Kelley:** Writing – review & editing, Visualization, Resources, Methodology, Investigation, Formal analysis. **Jeroen Vanoirbeek:** Writing – review & editing, Writing – original draft, Visualization, Resources, Methodology, Conceptualization.

## Declaration of competing interest

The authors declare that they have no known competing financial interests or personal relationships that could have appeared to influence the work reported in this paper.

## Data Availability

Data will be made available on request.

## Appendix A Supplementary data

This article has an online data supplement, that is accessible from this issue's table of contents online at **the journal website.**
