# Supplementary material for: Intermittent ozone inhalation during house dust mite-induced sensitization primes for adverse asthma phenotype
Source: Redox Biol. 2024 Aug 28;76:103330. doi: 10.1016/j.redox.2024.103330 (PMC11407077; doi:10.1016/j.redox.2024.103330)
Supplement: Multimedia component 2 [file mmc2.pptx]

## Slide 1
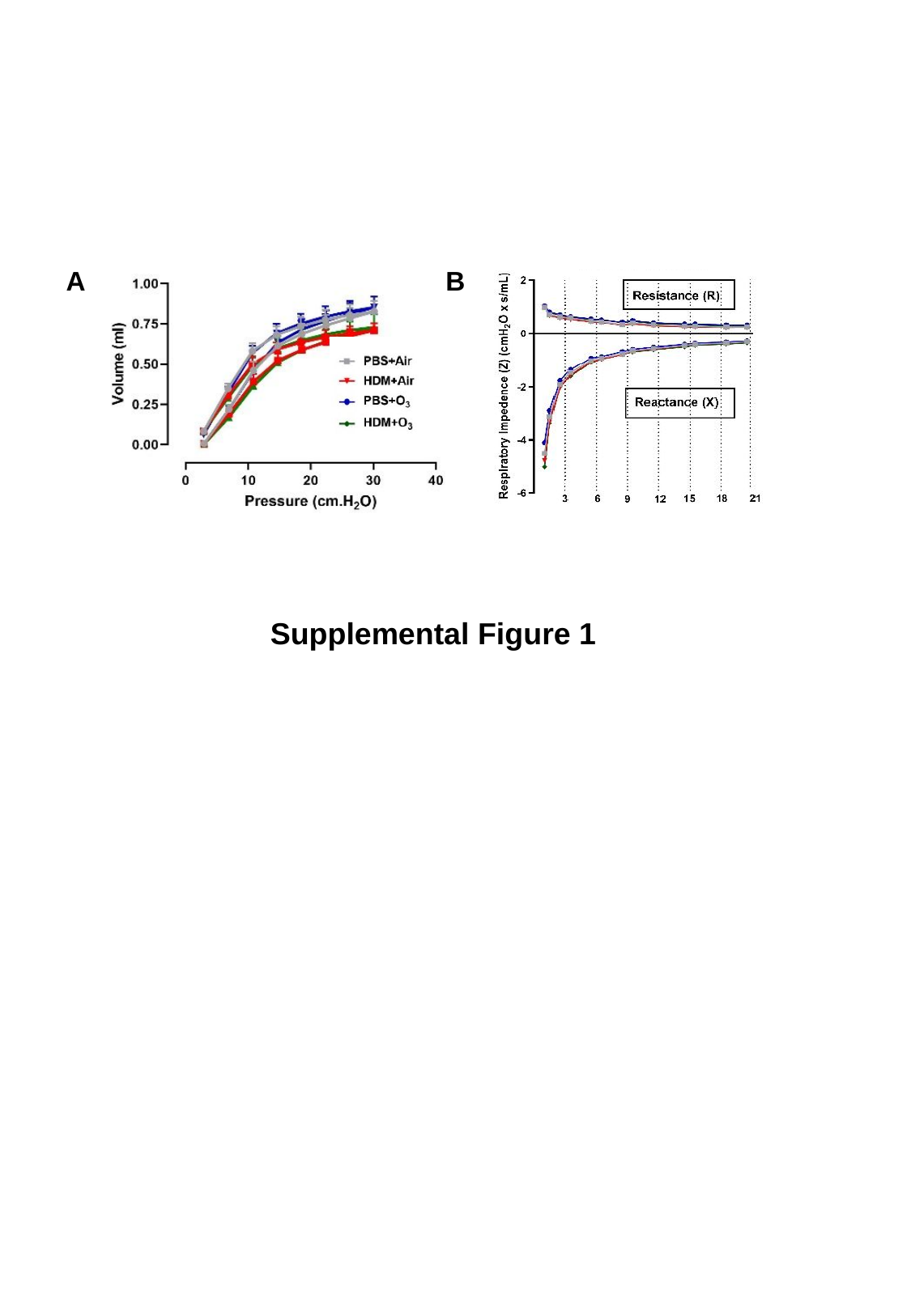

A
B
Supplemental Figure 1

## Slide 2
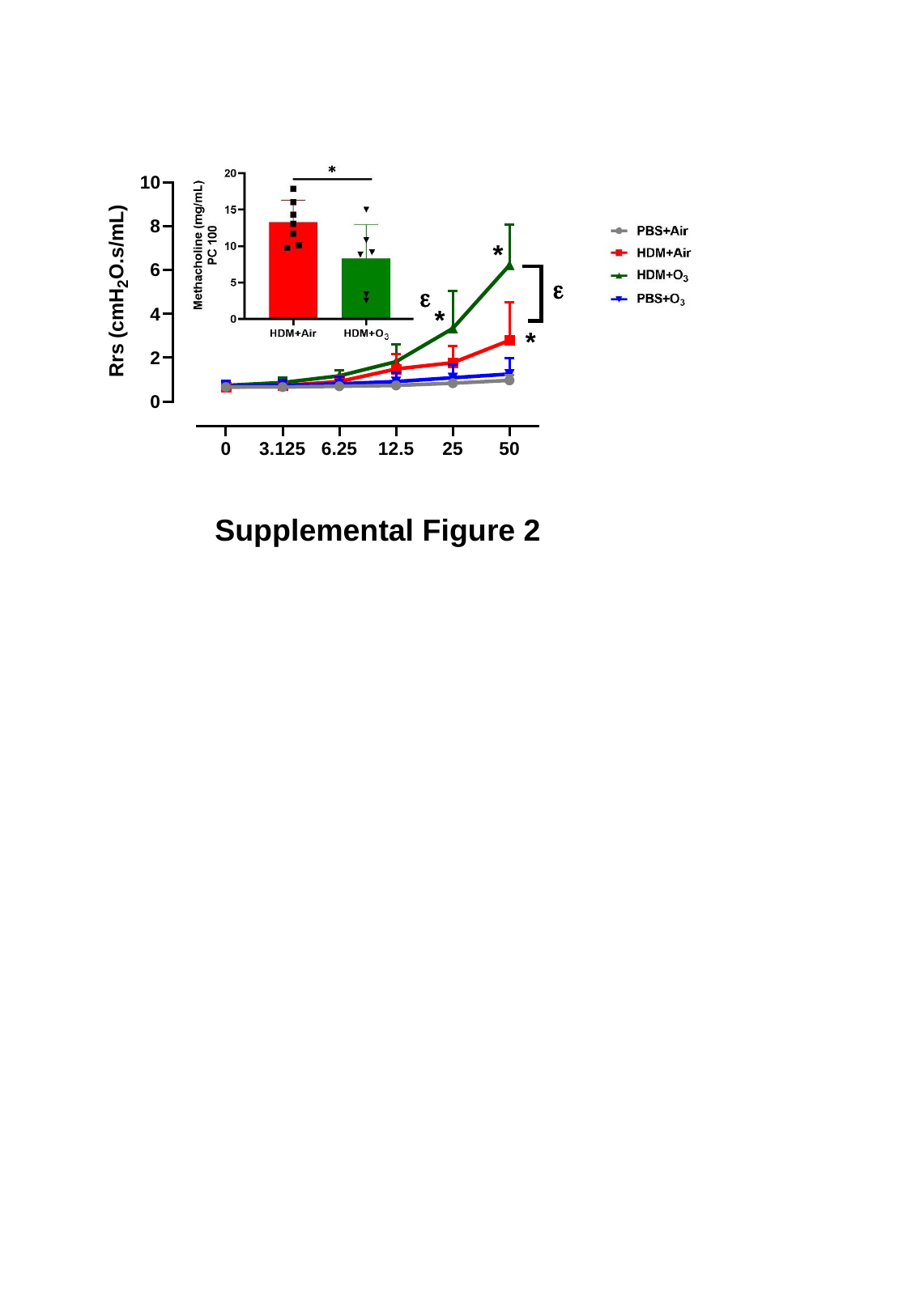

Supplemental Figure 2

## Slide 3
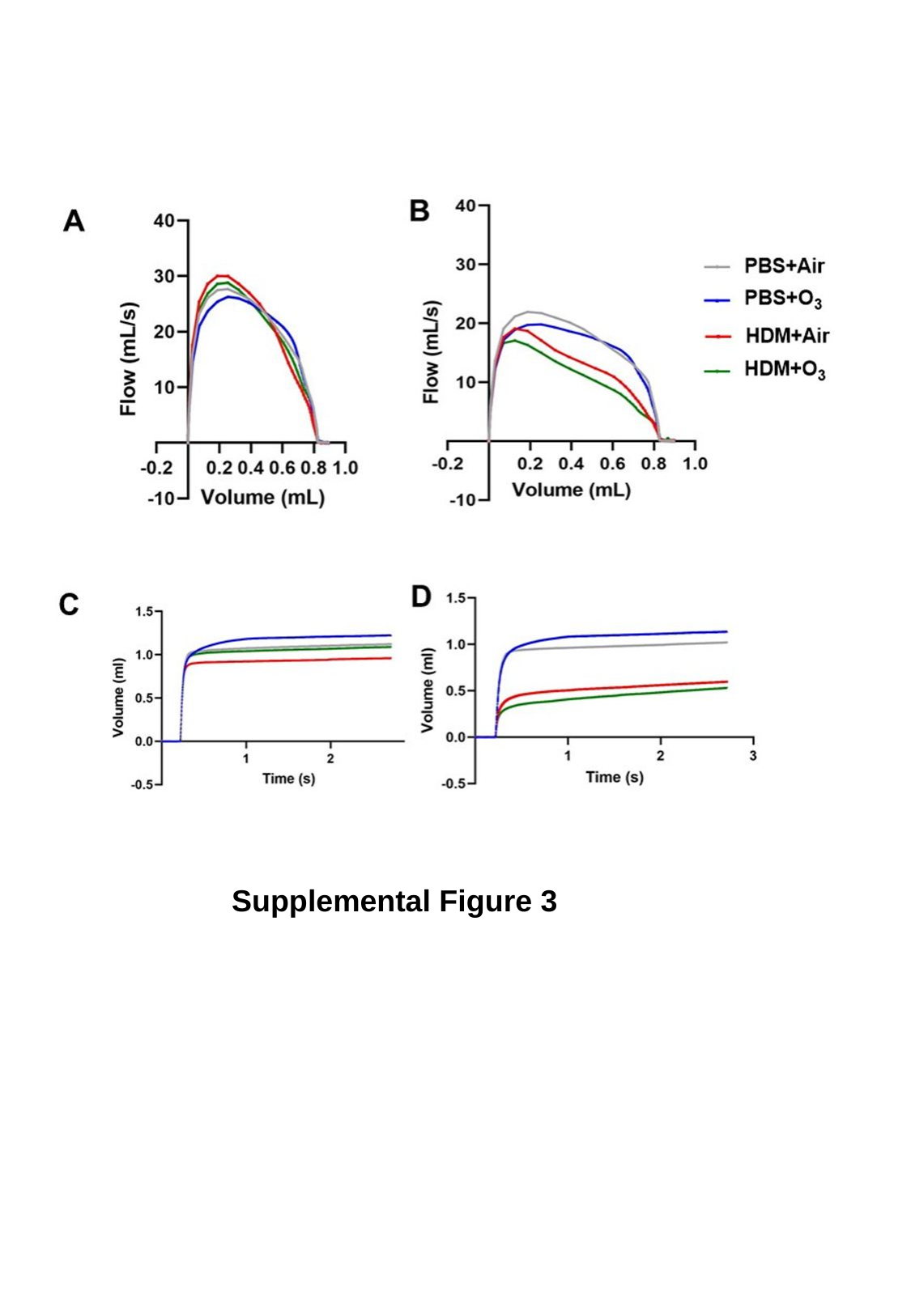

Supplemental Figure 3

## Slide 4
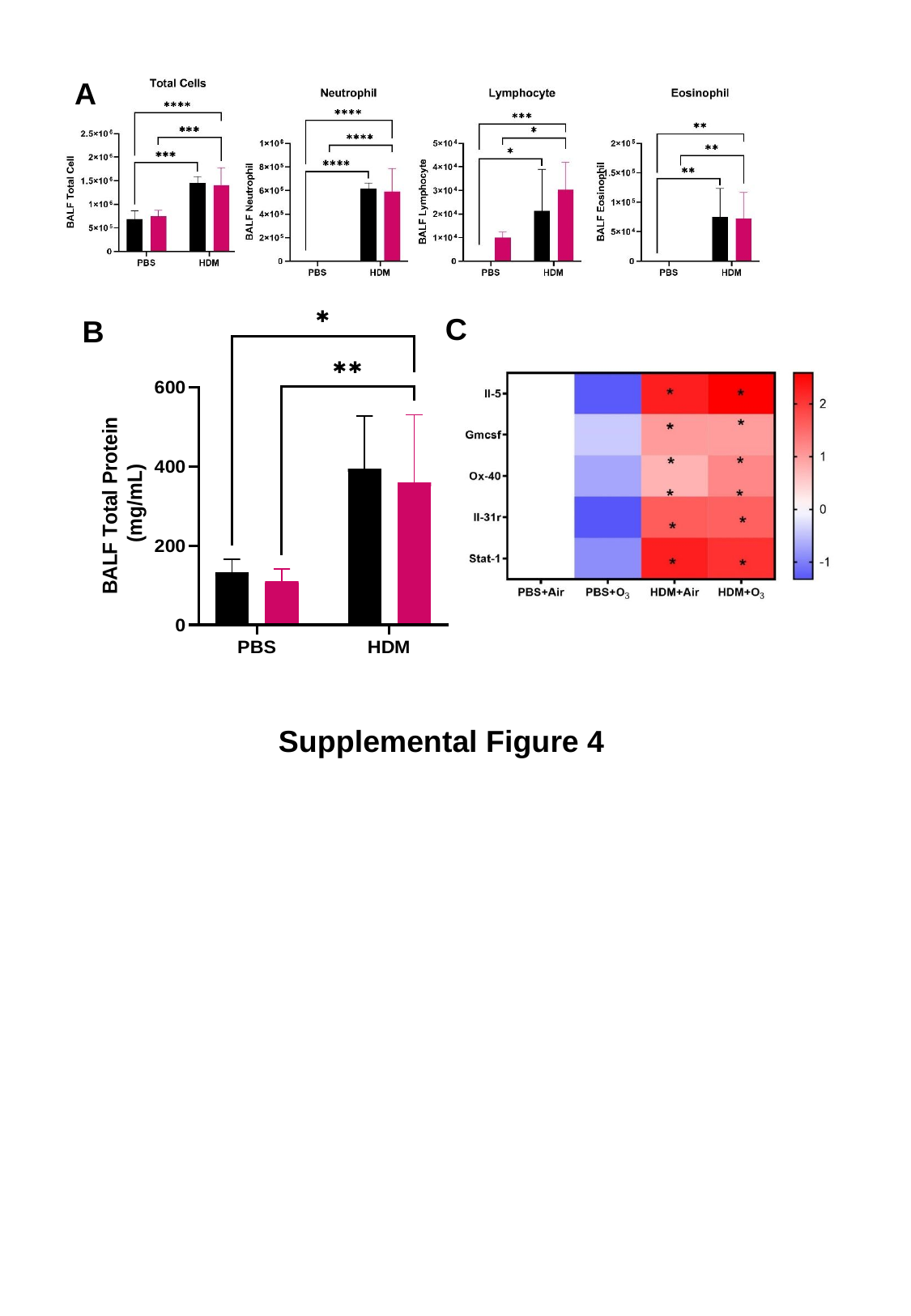

A
C
B
Supplemental Figure 4

## Slide 5
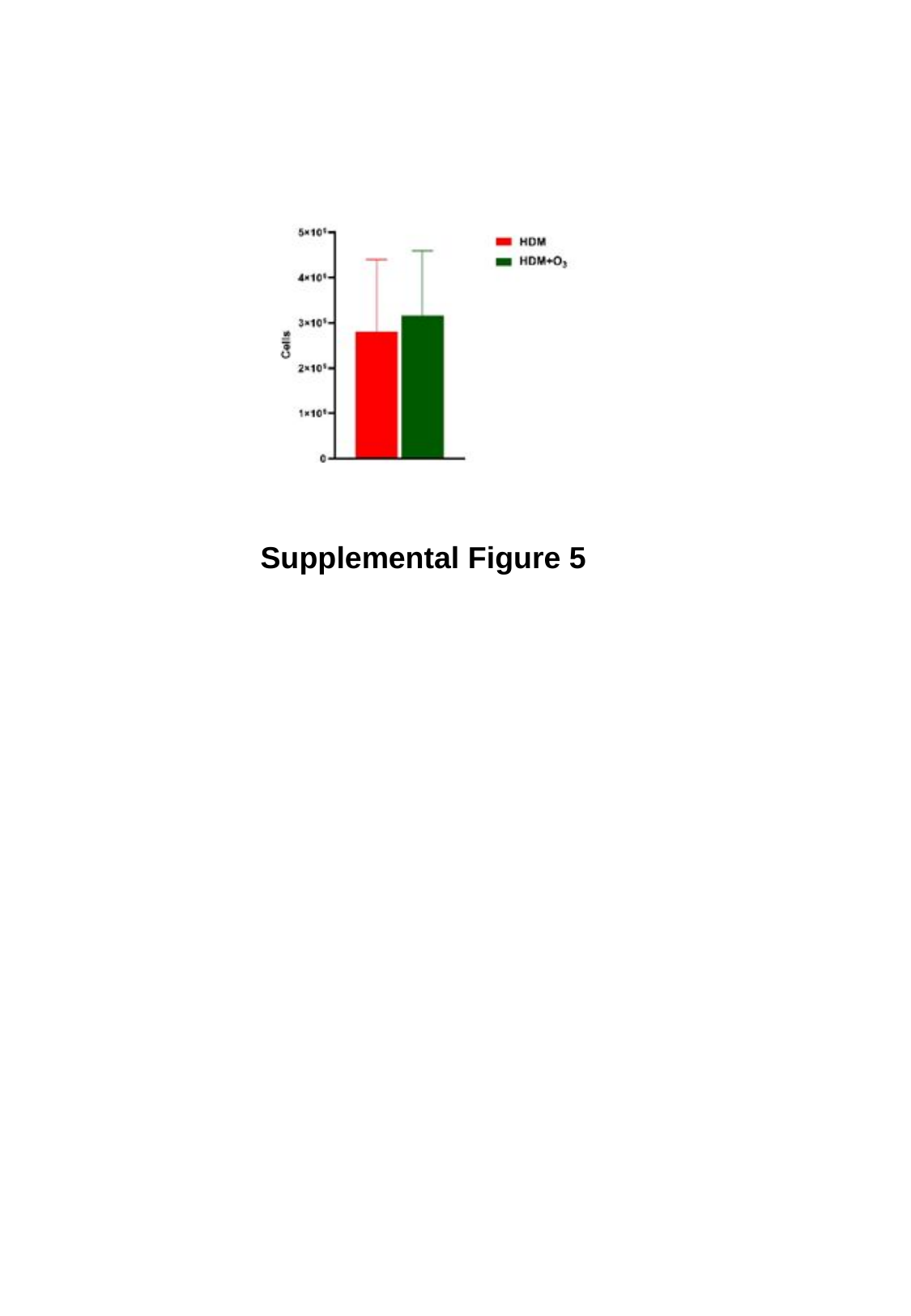

Supplemental Figure 5

## Slide 6
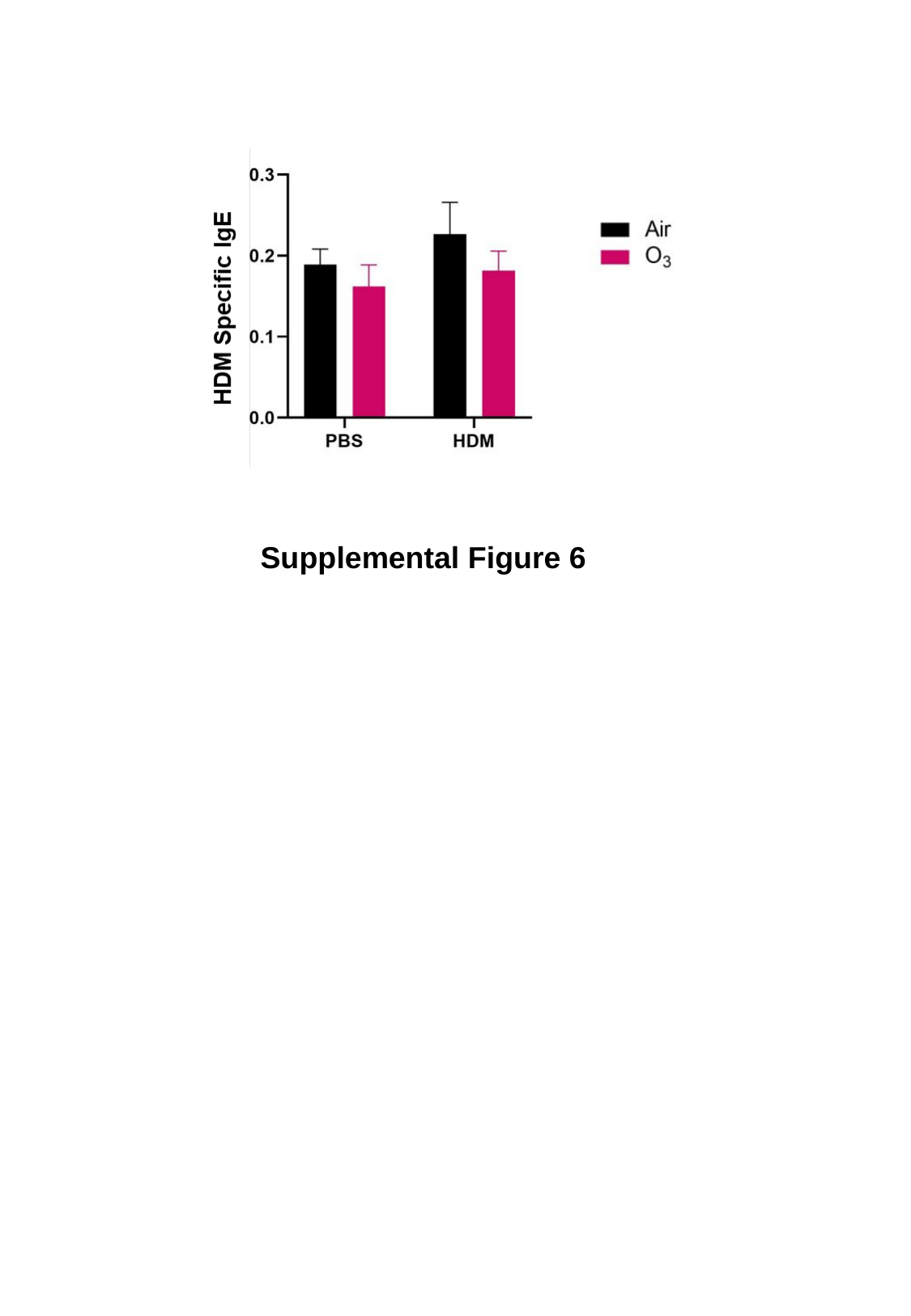

Supplemental Figure 6

## Slide 7
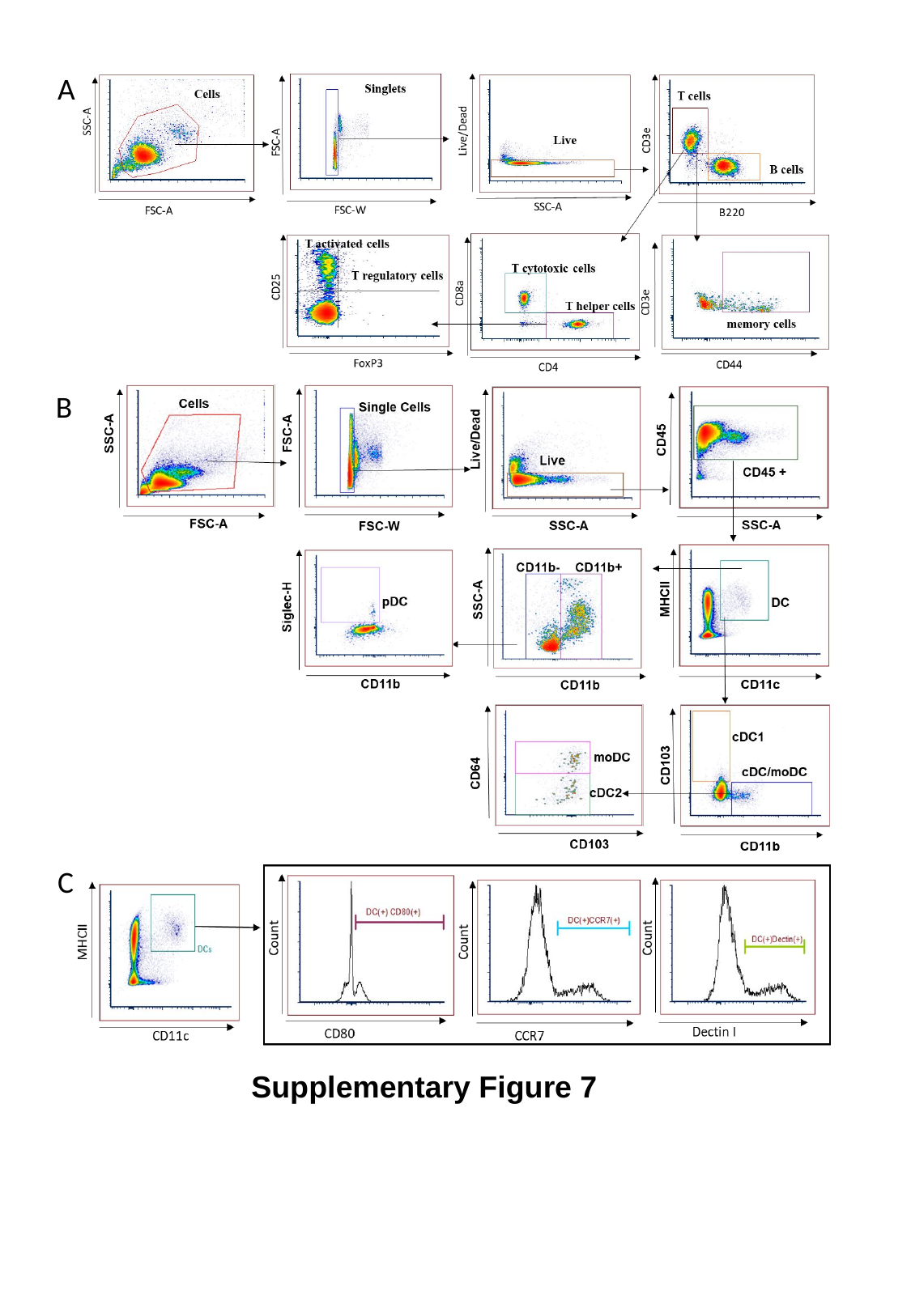

A
B
C
Supplementary Figure 7

## Slide 8
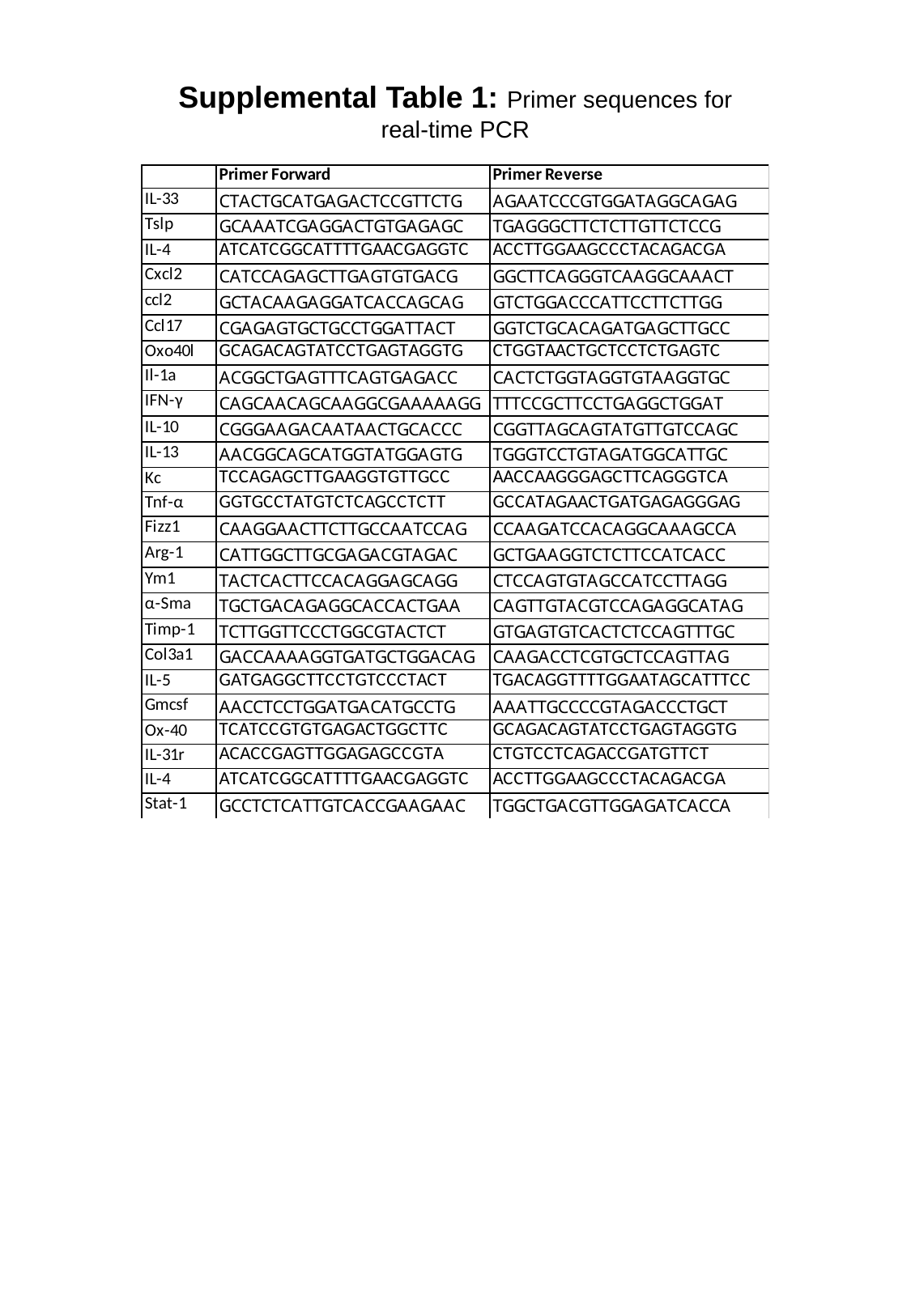

Supplemental Table 1: Primer sequences for real-time PCR

## Slide 9
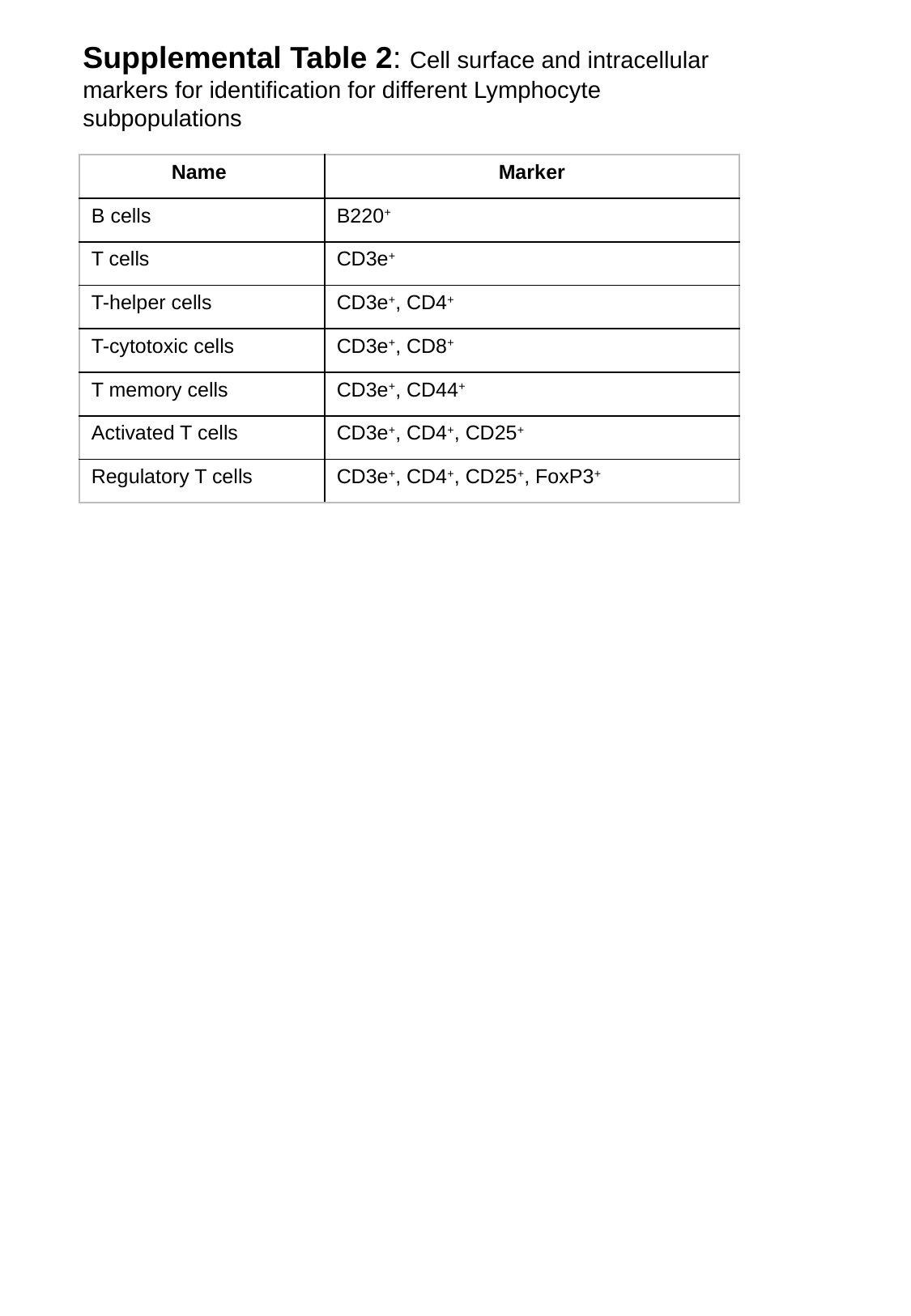

Supplemental Table 2: Cell surface and intracellular markers for identification for different Lymphocyte subpopulations
| Name | Marker |
| --- | --- |
| B cells | B220+ |
| T cells | CD3e+ |
| T-helper cells | CD3e+, CD4+ |
| T-cytotoxic cells | CD3e+, CD8+ |
| T memory cells | CD3e+, CD44+ |
| Activated T cells | CD3e+, CD4+, CD25+ |
| Regulatory T cells | CD3e+, CD4+, CD25+, FoxP3+ |

## Slide 10
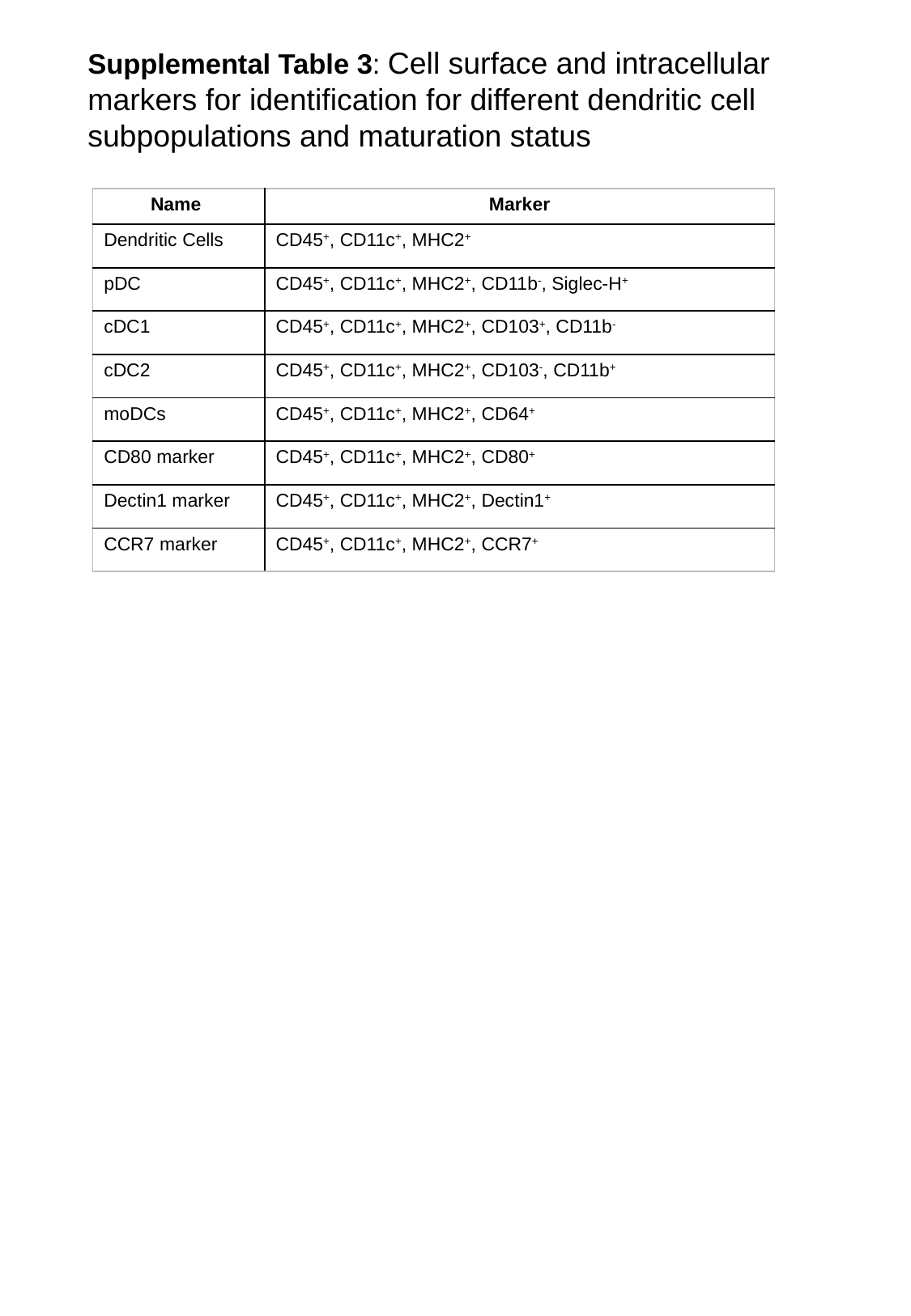

Supplemental Table 3: Cell surface and intracellular markers for identification for different dendritic cell subpopulations and maturation status
| Name | Marker |
| --- | --- |
| Dendritic Cells | CD45+, CD11c+, MHC2+ |
| pDC | CD45+, CD11c+, MHC2+, CD11b-, Siglec-H+ |
| cDC1 | CD45+, CD11c+, MHC2+, CD103+, CD11b- |
| cDC2 | CD45+, CD11c+, MHC2+, CD103-, CD11b+ |
| moDCs | CD45+, CD11c+, MHC2+, CD64+ |
| CD80 marker | CD45+, CD11c+, MHC2+, CD80+ |
| Dectin1 marker | CD45+, CD11c+, MHC2+, Dectin1+ |
| CCR7 marker | CD45+, CD11c+, MHC2+, CCR7+ |
