# Supplementary material for: Intermittent ozone inhalation during house dust mite-induced sensitization primes for adverse asthma phenotype
Source: Redox Biol. 2024 Aug 28;76:103330. doi: 10.1016/j.redox.2024.103330 (PMC11407077; doi:10.1016/j.redox.2024.103330)
Supplement: Multimedia component 3 [file mmc3.docx]

**Supplemental Material**

**Intermittent ozone inhalation during house dust mite-induced allergic sensitization primes for adverse asthma phenotype**

**Supplementary Figure and Table Legends.**

**Supplementary Figure 1:** A) Lung Pressure volume loops at baseline B) Respiratory impedance measured using the forced oscillation technique with pseudo-random oscillations over a range of 1–20.5 Hz depicting the real (i.e., resistance) and imaginary (i.e., reactance) part of the impedance at baseline. Data are presented as mean ± SD of n = 5–9 mice per group

**Supplementary Figure 2:** Total Respiratory resistance (Rrs) at baseline (inset) and after methacholine provocation. two-way analysis of variance (ANOVA) followed by Tukey's post hoc test. Data are presented as mean ± SD of n = 5–9 mice per group .*P ≤ 0.05 vs PBS+Air, ε P ≤ 0.05 between HDM+Air and HDM+O_3_ that methacholine concentration.

**Supplementary Figure 3:** A) Lung flow-volume curve at baseline B) Lung flow-volume curve at the highest dose of methacholine provocation C) Lung time-volume curve at baseline D) Lung time-volume curve at the highest dose of methacholine provocation (50mg/mL). Data are presented as mean. n = 5–9 mice per group

**Supplementary Figure 4:** A) Broncho-alveolar lavage total Cell, lymphocyte, eosinophil and neutrophil count B) Broncho-alveolar lavage total protein C) Lung tissue real-time PCR mRNA expression. n = 5–7 mice per group and analyzed by two-way analysis of variance (ANOVA) followed by Tukey's post hoc test. PCR values are presented as Log 2-fold change values. * represents significantly different from PBS+Air_._

**Supplementary Figure 5:** Supplemental Figure 5. Absolute Th cell numbers in HDM and HDM+O_3_ treated mice. The experimental design and other details are the same as in Figure 5.

**Supplementary Figure 6:** A) HDM-specific serum IgE (1:20000). n = 5–7 mice per group and analyzed by one-way analysis of variance (ANOVA) followed by Tukey's post hoc test.

**Supplementary Figure 7**: Gating strategy for analyzing immune cell phenotype **A)** Lymphocyte cells (T, B, memory cells, T _helper_, T _cytotoxic_, activated T and regulatory T cells) gating strategy, **B)** Gating strategy for dendritic cells (cDC1, cDC2, moDC, and pDC), **C)** Gating strategy for cell activation and maturation markers.
